# Changes of Androstenone Concentrations in Saliva of Boars with Age

**DOI:** 10.3390/ani12020157

**Published:** 2022-01-10

**Authors:** Kamila Pokorná, Jaroslav Čítek, Petr Doležal, Martyna Małopolska, Mirosłav Tyra, Monika Okrouhlá, Kateřina Zadinová, Michal Šprysl, Nicole Lebedová, Roman Stupka

**Affiliations:** 1 Department of Agro-Environmental Chemistry and Plant Nutrition, Faculty of Agrobiology, Food and Natural Resources, Czech University of Life Sciences Prague, Kamýcká 129, 165 00 Prague, Czech Republic; citek@af.czu.cz (J.Č.); dolezal1@af.czu.cz (P.D.); okrouhla@af.czu.cz (M.O.); zadinova@af.czu.cz (K.Z.); sprysl@af.czu.cz (M.Š.); lebedova.nicole@vuzv.cz (N.L.); stupka@af.czu.cz (R.S.); 2 Department of Animal Science, Faculty of Agrobiology, Food and Natural Resources, Czech University of Life Sciences in Prague, Kamýcká 129, 165 00 Prague, Czech Republic; martyna.malopolska@iz.edu.pl (M.M.); miroslaw.tyra@iz.edu.pl (M.T.)

**Keywords:** boar taint, entire male pig, salivary gland, steroid hormone, welfare

## Abstract

**Simple Summary:**

Pork is one of the most consumed meats worldwide. In many countries, pork is produced only from gilts or barrows because of the presence of boar taint in boar’s meat. Boar taint is an unpleasant odour, which is present in the meat and fat of boars. This odour is related to the increasing age and body weight of boars. The absence of boar taint in the final product can be achieved by castration of boars. Regarding the welfare of the animals, it would be appropriate to find a non-invasive, reliable, and working method for the determination of boar taint during the fattening of boars. Androstenone, which is one of the main compounds responsible for boar taint, can be determined from saliva. This method appears to be a hopeful way to predict individuals with a high level of this compound. Based on the current knowledge, the early slaughter of boars could be a suitable and functional step to avoid boar taint in the meat or fat, respectively. This is a pilot study that is focused on the non-invasive determination of boar taint on live animals. In the future, this method could be simplified and adjusted for use in farm conditions.

**Abstract:**

With the increasing age of boars, the possibility of androstenone (5α-androst-16-en-3-one (AND), 5α-androst-16-en-3α-ol (α-AND), and 5α-androst-16-en-3β-ol (β-AND)) occurrence increases as well. The aim of this study was to evaluate concentrations of androstenone compounds in the saliva of boars concerning the age of animals. In total, 72 boars were evaluated (24 boars per replication). The effect of age (three different ages—152, 163, and 172 days) was observed, and (Landrace × Large White) × Pietrain genotypes were used. Chemical analysis of saliva samples was conducted by multidimensional gas chromatography/mass spectrometry (MDGC/MS). Salivary α-AND increased with age (*p* < 0.05), and positive correlations were found between age and submaxillary salivary gland weight (*p* < 0.05), age, and salivary AND concentration (*p* < 0.05), body weight and submaxillary salivary gland weight (*p* < 0.05), submaxillary salivary gland weight and salivary β-AND concentration (*p* < 0.05), as well as submaxillary salivary gland weight and total salivary 5α-androstenone (AND total) concentration (*p* < 0.001). Nowadays, animal welfare is becoming a more and more discussed topic, and pig breeding is not an exception. Specifically, the castration ban is a current issue, so it is very important to know as much as possible about compounds responsible for boar taint. Androstenone’s appearance in boars’ saliva could be one of the most important precursors for future early detection of boar taint.

## 1. Introduction

Boar’s bodies go through many changes with age to ensure their reproductive ability. These include changes in anatomy, behaviour, and, of course, hormone production [1]. In relation to pork meat production, very important are hormonal changes. As boars age, there is a growing possibility for heightened production of compounds responsible for boar taint [2]. Boar taint is an odour or taste that can be evident during the cooking or eating of pork products derived from noncastrated male pigs [3,4,5]. One of these compounds is the hormone androstenone, which causes an unpleasant “urine-like” odour in pork. Androstenone is a steroid hormone produced by Leydig cells [6,7,8] together with testosterone. It is stored in adipose tissue or secreted by the salivary glands into the saliva. Saliva is produced in boars by three main pairs of salivary glands: the parotid, submaxillary, and sublingual glands [9]. It is predominantly the submaxillary salivary glands that are associated with the metabolism of the boar taint compound androstenone. These salivary glands do not contain the andien-β synthetase system that is necessary for the synthesis of 5,16-androstadien-3β-ol from pregnenolone. Therefore, they are not able to synthesise steroids from this precursor [10], but they are able to metabolise all steroids transported to them from circulating blood [11]. Boars’ submaxillary salivary glands possess the enzyme system needed for the transformation of 5,16-androstadien-3β-ol to 5α-androst-16-en-3α-ol and 5α-androst-16-en-3β-ol. It is this ability that enables a boar’s saliva to contain high concentrations of 5α-androst-16-en-3-one, 5α-androst-16-en-3α-ol, and 5α-androst-16-en-3β-ol during sexual excitement [12,13]. The concentration of androstenone in the saliva of boars is primarily dependent upon the onset of puberty, it is related to age and live weight of boars, and it is greatly individual [14,15]. During puberty, levels of steroid hormones begin to increase, causing changes in sexual and aggressive behaviour in boars [16]. Levels of androstenone in the saliva of boars are related to the basic manifestations of sexual behaviour. Its “urine-like” odour makes androstenone in saliva a pheromone [17]. The presence of androstenone in saliva results in the lordosis reflex in a sow when she is in oestrus. This signals when is the right time for sows to mate or be inseminated. Therefore, the role of androstenone in saliva is very important. Androstenone pathways in salivary glands are known [18], but the question remains as to how androstenone’s concentration in the saliva of boars increases with age. Positive correlations between the weight of submaxillary salivary gland and age, between the weight of testis and the weight of submaxillary salivary glands, and between the concentration of 5α-androst-16-en-3-one and the weight of submaxillary salivary glands were observed [19]. However, no results about the levels of androstenone in the saliva of boars were reported. With a goal of improving animal welfare, researchers most often rely on obtaining saliva from animals in order to analyse specific compounds. Collecting saliva from boars for analyses is a non-invasive method of the future. The aim of this study was to evaluate the relationship between androstenone concentrations in boars’ saliva and their age. Based upon the literature, it was hypothesised that, due to the effective enzyme system in their submaxillary salivary glands, there should occur increasing levels of 5α-androst-16-en-3-one, 5α-androst-16-en-3α-ol, and 5α-androst-16-en-3β-ol in saliva in relation to age.

## 2. Materials and Methods

All experimental procedures were approved by the Ethics Committee of the Central Commission for Animal Welfare at the Ministry of Education Youth and Sports of the Czech Republic (Prague, Czech Republic) and were carried out in accordance with Directive 2010/63/EU regarding animal experiments and Local Ethics Commission, case number 13/2018. The experiment was conducted in the Demonstration and Experimental Centre at the Czech University of Life Sciences Prague (CZ11760061/01).

### 2.1. Animals

In the present study, three replications of the experiment design were used. We evaluated 72 boars (24 boars per replication). Moreover, we studied three different ages of boars (8 boars per age). Boars ((Landrace × Large White) × Pietrain) were housed individually under identical conditions in the Demonstration and Experimental Centre of the Czech University of Life Sciences Prague. In the Demonstration and Experimental Centre, standard EU microclimatic conditions were secured, which remove the effects of the season. The average floor area per boar was a minimum of 1 m^2^. Boars were fed ad libitum with complete compound feed. The first group were slaughtered at the age of 152 days and average live weight of 105.20 kg (SEM = 2.769 kg), the second group at the age of 163 days and average live weight of 119.30 kg (SEM = 1.531 kg), and the third group at the age of 172 days and average live weight of 133.50 kg (SEM = 4.394 kg). The age of slaughtering was chosen based on reaching the standard slaughter weight of boars and based on the average age of slaughtered pigs in the EU (150–180 days).

### 2.2. Post-Mortem Sample Collection

Samples of adipose tissue and submaxillary salivary glands were taken post-mortem for testing. Samples of adipose tissue were collected 24 h after slaughter in the area between the first and third cervical vertebrae. The weight of the average sample was 30 g. The samples were packed without skin and muscles and stored in a freezer at −80 °C. The analysis of the androstenone levels in the adipose tissue was performed using HPLC (Jasco LC-2000, Watrex Praha, s.r.o., Prague, Czech Republic) described by methodology [20]. To determine androstenone levels, an Agilent Eclipse XDB C18 column (5 μm, 150 × 4.60 mm ID) operated at 40 °C was used. The parameters of the mobile phase were: (A) tetrahydrofuran: acetonitrile: sodium phosphate buffer (25 mM): acetic acid (34: 23.8: 41.4: 0.8) and (B) methanol. Fluorescence detection was performed with excitation at 346 nm and emission at 521 nm. The standard calibration curve was used to determine the content of androstenone in the samples. The observations were evaluated using the programme ChromNAV (ver. 1.18.04.) [20]. ChromNAV (ver. 1.18.04.)

### 2.3. Saliva Sample Collection

Saliva was collected on cotton wool tampons one week before slaughtering. It was ensured that the boars had no access to water or food for approximately 15 min before saliva collection. Tampons with absorbed saliva (3 mL) were placed into individual plastic bags, and saliva was pressed out from the tampons. After collection, saliva was stored in glass tubes at −80 °C to ensure the stability of androstenone [21,22].

### 2.4. MDGC-MS Analyses

Saliva samples were processed in the laboratories of the Department of Animal Science at the Czech University of Life Sciences Prague. The chemical analysis was conducted by multidimensional gas chromatography/mass spectrometry (MDGC/MS). The MDGC/MS set up included two GC-2010 ovens (Shimadzu, Kyoto, Japan) and columns with two different stationary phases. The compounds 5α-androst-16-en-3-one (AND), 5α-androst-16-en-3α-ol (α-AND), and 5α-androst-16-en-3β-ol (β-AND) were determined from the saliva while following the modified methodology described by Dehnhard et al. [23]. The samples of saliva were analysed using a 50 m FS-Supreme-5 ms capillary column. Ultrapure helium was used as a carrier gas, with a column head pressure setting of 41 kPa. The injection temperature was 300 °C. The MS acquisition was performed in SIM by monitoring the ions m/z 272 for 5α-androst-16-en-3-one, m/z 274 for 5α-androst-16-en-3α-ol (α-AND), and 5α-androst-16-en-3β-ol (β-AND) and m/z 202 for 5α-androst-3-one (internal standard). A calibration line and constant amount of internal standard were used for quantification [23].

### 2.5. Statistical Analysis

Statistical evaluation was performed in SAS (Statistical Analysis System, version 9.4, 2012; SAS Institute, Cary, NC, USA). A generalised linear model procedure was used for evaluating individual effects (i.e., the effect of age on the submaxillary salivary gland’s weight, effect of age on salivary AND concentration, effect of age on salivary α-AND concentration, effect of age on salivary β-AND concentration, and effect of age on AND concentration in adipose tissue). The Pearson correlation coefficient was used for evaluating correlation.

The following indicators were calculated and evaluated: least-squares means, standard errors of the means, and *p*-values (while setting statistical significance at α ≤ 0.05).

## 3. Results

### 3.1. Effect of Age on Androstenone Concentrations in Saliva and Adipose Tissue of Boars

Statistically significant differences among groups were observed and are presented in Table 1. The weight of submaxillary salivary glands increases with age (*p* < 0.05). The concentration of salivary AND increases with age (*p* < 0.05). The highest concentration of AND was in the oldest group of boars. The increasing concentration of salivary α-AND also was determined in relationship with the increasing age of boars. The highest concentration of α-AND was found in the oldest group of boars. The oldest boars reached the highest levels of androstenone concentration, and statistically significant differences among groups were found in concentrations of total salivary AND (*p* < 0.05). The level of total salivary AND concentration increased with boars’ age. No differences among groups were observed in levels of AND in adipose tissue.

### 3.2. The Relationships between Studied Indicators

Table 2 describes the relationships among the various indicators. Positive correlations were observed between most indicators. Strong positive correlations were observed between age and submaxillary salivary gland weight (*p* < 0.001), age and salivary AND concentration (*p* < 0.001), body weight and submaxillary salivary gland weight (*p* < 0.001), submaxillary salivary gland weight and salivary β-AND concentration (*p* < 0.01), as well as submaxillary salivary gland weight and total salivary AND concentration (*p* < 0.001).

## 4. Discussion

The mean level of salivary α-AND of German Landrace boars was 0.18 μg/mL [23]. In our study, the mean measured concentrations of α-AND were 0.012 μg/mL for the youngest boars, 0.019 μg/mL for a middle group of boars, and 0.026 μg/mL for the oldest boars. The levels of salivary β-AND were more balanced between groups. The mean level of salivary β-AND of German Landrace was 0.02 μg/mL [23]. Additionally, in this study, the mean measured levels of β-AND were 0.017 μg/mL for the youngest boars, 0.022 μg/mL for a middle group, and 0.018 μg/mL for the oldest boars. The differences could be caused by using different breeds and hybrids with different seasonality in these studies. Walstra et al. [24] observed higher levels of androstenone in adipose tissue of pigs during the summer in most European countries. Next significant breed differences in 16-androstenes levels among Duroc, Hampshire, Landrace and Yorkshire were reported [25]. For the next evaluation, it would be good to compare groups of most used hybrid boars because of their routine use in fattening. According to Hurden et al. [26], in testis, there predominates a reduction in AND to its 3β isomer so that the saliva contains lower levels of β-AND. Booth [27] had reported that the concentration of β-AND exceeded that of α-AND in post-pubertal testes and that α-AND is the dominant component in submaxillary salivary glands at all ages. This was confirmed by our results only in one case, as the oldest boars had higher levels of α-AND in saliva than of the β isomer.

Our results confirmed previous studies, which showed the concentration of 5α-androstenone has a tendency to increase as boars become older and heavier [28]. The oldest boars reached the highest levels of androstenone concentration, and statistically significant differences among groups were found in concentrations of total salivary AND (*p* < 0.05). Booth et al. [19] had confirmed a positive correlation between 5α-androstenone concentration in the salivary gland and the submaxillary salivary gland weight. The lowest levels of androstenone in salivary glands are related to the smallest salivary glands. Similarly, positive correlations between age and submaxillary salivary gland weight (*p* < 0.001), age, and salivary AND concentration (*p* < 0.001) were observed. Moreover, positive relationships were found between body weight and submaxillary salivary gland weight (*p* < 0.001), submaxillary salivary gland weight and salivary β-AND concentration (*p* < 0.01), as well as submaxillary salivary gland weight and total salivary 5α-androstenone concentration (*p* < 0.001). Babol et al. [29] further stated positive correlations among 16-androstene steroids and synthesis of oestrogens and androgens. Booth et al. [19] had reported positive correlations between age and submaxillary salivary gland weight and stated that the submaxillary salivary gland’s weight increases with body weight and age, which is consistent with our results. Babol et al. [30] observed positive correlations between the concentrations of 16-androstene steroids in salivary glands and androstenone in fat. Positive correlations of boar taint compounds between different types of tissues offer the possibility to expand the field of research. For the future, it would be appropriate to measure androstenone as well as skatole for boar taint detection. More studies need to be executed to completely understand this problem.

## 5. Conclusions

In terms of animal welfare requirements and the possibility of a ban on castration, it is very important to know as much as possible about compounds responsible for boar taint. To recapitulate, salivary 5α-androstenone increases with boar age, and its appearance in boars’ saliva could be one of the most important precursors for future early detection of boar taint. We found the possibility of using this non-invasive method to examine androstenone concentration in boars’ saliva, which could improve animal welfare in fattening and meet both the consumers’ and pork producers’ expectations.

## Figures and Tables

**Table 1:** Comparison of groups of boars as of slaughter date for levels of AND, α-AND, and β-AND.

Scheme 152	152 (n = 24)		163 (n = 24)		172 (n = 24)	
	Mean	SEM ^1^	Mean	SEM ^1^	Mean	SEM ^1^
Live weight (kg)	105.20	2.769	119.30	1.531	133.50	4.394
SMG ^2^ weight (g)	23.30 ^B^	2.614	27.97 ^AB^	6.405	41.70 ^A^	4.800
Salivary AND ^3^ (μg/mL)	0.001 ^B^	0.001	0.002 ^AB^	0.002	0.009 ^A^	0.005
Salivary α-AND ^4^ (μg/mL)	0.012	0.006	0.019	0.007	0.026	0.032
Salivary β-AND ^5^ (μg/mL)	0.017	0.002	0.022	0.008	0.018	0.004
Salivary AND ^6^ total (μg/mL)	0.030 ^B^	0.009	0.043 ^AB^	0.012	0.053 ^A^	0.037
AND ^3^ in adipose tissue (μg/g)	3.678	2.327	3.491	1.870	4.974	1.703

^1^ SEM—standard error of the mean. Superscripts (AB) indicate statistically significant differences (with *p* ≤ 0.05). ^2^ SMG—submaxillary gland. ^3^ AND—5α-androst-16-en-3-one. ^4^ α-AND—5α-androst-16-en-3α-ol. ^5^ β-AND—5α-androst-16-en-3β-ol. ^6^ AND total—5α-androstenone total.

**Table 2:** Correlation coefficients between studied indicators.

Item	Age	Body Weight	SMG ^1^ Weight	Salivary AND ^2^	Salivary α-AND ^3^	Salivary β-AND ^4^	Salivary AND Total ^5^
Age	1.000						
	0.000						
Body weight	0.816	1.000					
	0.001	0.000					
SMG ^1^ weight	0.902	0.872	1.000				
	0.001	0.001	0.000				
Salivary AND ^2^	0.887	0.650	0.771	1.000			
	0.001	0.022	0.009	0.000			
Salivary α-AND ^3^	0.559	0.460	0.747	0.511	1.000		
	0.059	0.133	0.013	0.090	0.000		
Salivary β-AND ^4^	0.358	0.404	0.857	0.341	0.468	1.000	
	0.254	0.193	0.002	0.279	0.125	0.000	
Salivary AND total ^5^	0.695	0.575	0.882	0.682	0.959	0.628	1.000
	0.012	0.050	0.001	0.015	0.001	0.029	0.000
AND ^2^ in adipose tissue	0.328	0.617	0.274	0.263	−0.200	0.024	−0.083
	0.298	0.033	0.443	0.410	0.534	0.941	0.797

^1^ SMG—submaxillary gland. ^2^ AND—5α-androst-16-en-3-one. ^3^ α-AND—5α-androst-16-en-3α-ol. ^4^ β-AND—5α-androst-16-en-3β-ol. ^5^ AND total—5α-androstenone total.

## Data Availability

The data presented in this study are available on a request from the corresponding author.
